# Immunity, gut microbiota and infection

**DOI:** 10.1186/1824-7288-40-S1-A12

**Published:** 2014-08-11

**Authors:** Susanna Esposito

**Affiliations:** 1 Pediatric Highly Intensive Care Unit, Department of Pathophysiology and Transplantation, Università degli Studi di Milano, Fondazione IRCCS Ca’ Granda Ospedale Maggiore Policlinico, Milan, Italy

## 

Recurrent infections are very common in children and a major challenge for pediatricians. They affect the children’s quality of life, cause absences from school and lost parental working days, and repeated medical examinations, hospital admissions as well as antibiotic therapies lead to high costs for society. Given their prevalence and clinical importance, various prevention strategies have been developed.

Some of these strategies have considered that innate and adaptive immunity are strictly related with gut microbiota and interact in the modulation of host’s reactivity to infections. Variations in gut microbiota related to age, nutrition or underlying disease may influence immune system defenses against viruses and bacteria. Furthermore, disturbed gut colonization patterns are proposed to be associated with the development of allergic disease. This explains why probiotics represent an important option in the prophylaxis and the management of recurrent infectious diseases as well as allergic diseases. However, the incomplete understanding of what constitutes a healthy gut microbiota that promotes tolerance, remains a challenge. Further understanding of gut microbial functions may pave the way for more effective prevention and treatment strategies.

Another approach used for prevention of recurrent infections is represented by the administration of immunostimulants: i.e. molecules of bacterial or synthetic origin that interact with immunological mechanisms *in vitro* and *in vivo*. Pidotimod (3-L-pyroglutamil-L-thiaziolidine-4-carboxylic acid) is a synthetic dipeptide which stimulates the phagocytic activity of polymorphonuclear leukocytes, enhances the killing activity of human alveolar macrophages, increases the cytotoxic activity of natural killer cells, and stimulates cytokine production. It also influences the maturation and activity of dendritic cells by increasing the expression of the key surface markers that help to trigger T cell activation and increased IL-12 production. The majority of studies have shown that the number of infections decreases after pidotimod treatment, but they are affected by some methodological weaknesses. Further studies are urgently needed to confirm the pediatric population that should have the greatest benefit with the administration of immunostimulants.

More studies are needed to identify the most promising probiotic strains and study populations, and to evaluate the mechanisms behind the possible effects of probiotics on OM.

